# Auricular acupressure for constipation in adults: a systematic review and meta-analysis

**DOI:** 10.3389/fphys.2023.1257660

**Published:** 2023-10-16

**Authors:** Ze-Fei Jiang, Guang Liu, Xiao-Xiang Sun, Na Zhi, Xue-Mei Li, Ran Sun, Hong Zhang

**Affiliations:** Acupuncture and Tuina School, Chengdu University of Traditional Chinese Medicine, Chengdu, Sichuan, China

**Keywords:** auricular acupressure, constipation, effectiveness, meta-analysis, systematic review

## Abstract

**Introduction:** Auricular acupressure (AA) has been widely utilized in the management of constipation, with several studies suggesting its efficacy in treating constipation patients. However, the safety and effectiveness of AA in constipation remain uncertain. Hence, the aim of this study was to assess the effectiveness and safety of AA for constipation.

**Methods and analysis:** A total of eight electronic databases and three clinical trial registration platforms were searched from their inception to April 2023 for randomized controlled trials (RCTs) of AA for constipation. The included studies were appraised for quality using the Cochrane Collaboration’s Risk of Bias Assessment tool. The quality of evidence was assessed by two independent reviewers employing the Grading of Recommendations Assessment, Development, and Evaluation System (GRADE) evaluation tool. Meta-analysis of data and assessment of publication bias were performed using RevMan 5.4 and STATA 13.0 software, respectively.

**Results:** This review included 34 randomized controlled trials conducted between 2007 and 2023, involving 2,465 participants. The findings of the study indicate that overall, AA is significantly associated with improved CSBMs (MD = 1.22, 95% CI [0.68, 1.77], *p* < 0.0001, I^2^ = 0%), BSF (MD = 0.72, 95%CI: [0.15,1.28], *p* = 0.01, I^2^ = 82%), CAS (MD = -3.28, 95%CI: [−5.95, −0.60], *p* = 0.02, I^2^ = 80%), responder rate (RR = 1.27, 95%CI: [1.16, 1.38], *p* < 0.00001, I^2^ = 79%), cure rate (RR = 1.84, 95% CI [1.56, 2.15], *p* < 0.00001, I^2^ = 0%), and PAC-QOL (MD = −2.73, 95% CI: [−3.41, −2.04], *p* < 0.00001, I^2^ = 98%) compared to the control group. However, no difference in PAC-SYM (MD = −0.15, 95%CI: [−0.38,0.07], *p* = 0.19, I^2^ = 67%) was found between the two groups. Additionally, there was no significant difference in adverse events (RR = 0.53, 95% CI: [0.24, 1.21], *p* = 0.13, I^2^ = 38%).

**Conclusion:** Based on the available evidence, auricular acupressure appears to be a potentially safe and effective intervention for managing constipation in adults. Nonetheless, the overall quality of evidence for the identified outcomes was assessed as low to very low, highlighting the need for additional high-quality randomized controlled trials to further validate these findings.

**Systematic Review Registration:** https://www.crd.york.ac.uk/prospero, identifier CRD42023425033.

## 1 Introduction

Constipation, characterized by difficult and infrequent bowel movements accompanied by symptoms like bloating and abdominal pain (Vriesman et al., 2020), has a higher prevalence in older adults due to age-related factors (Barberio et al., 2021). The estimated overall prevalence of constipation in older adults is 18.9% [95% CI (14.7%–23.9%)] (Salari et al., 2023). Among the available treatment options, saline laxatives have shown robust evidence of effectiveness (Serra et al., 2020). However, concerns persist regarding the long-term safety of this approach (Serra et al., 2020), as laxative misuse and abuse can lead to weight loss, electrolyte imbalances, laxative dependence, and visceral organ dysfunction (Daniali et al., 2020). Non-pharmacological interventions, such as dietary guidance, regular physical activity, and potty training, are considered the initial management strategy for constipation (Vriesman et al., 2020). Nevertheless, successful implementation of these interventions relies heavily on patient compliance and persistence. Therefore, it is crucial to develop an effective, safe, and feasible therapy to alleviate constipation.

Auricular acupressure (AA) is a non-invasive therapy rooted in Traditional Chinese Medicine (TCM) that involves applying pressure to specific acupoints on the ear using Vaccaria seeds or magnetic pellets. AA offers several advantages over other therapies, including affordability, painlessness, and high patient acceptance. Under the guidance of a doctor, patients can easily complete the treatment procedure. Each organ in the body has a reflex point on the outer ear’s surface, and acupoint stimulation aims to restore balance between yin and yang, harmonize the flow of vital energy (qi) and blood, and alleviate various ailments within the body (Zhu, 2014). The vagus nerve plays a crucial regulatory role in controlling smooth muscle contractions and glandular secretions in the intestines (Breit et al., 2018). AA may potentially have a similar effect on constipation as auricular vagal nerve stimulation (Shi et al., 2021; Zhang et al., 2021).

Several clinical studies have found it to be effective in treating constipation (Li et al., 2014; Shin and Park, 2018; Jung and Chang, 2020; Aminizadeh et al., 2023). To date, two systematic reviews (SRs) have been published demonstrating that AA can be used for the treatment of constipation in leukemia patients undergoing chemotherapy (Chen et al., 2018a; Jing et al., 2018a), however, both reviews included only five studies, and the overall quality of evidence was low. The safety and efficacy of AA on constipation are still unclear, hence a SR and meta-analysis regarding AA for constipation is necessary. Consequently, we aimed to evaluate the efficacy of AA for constipation through this meta-analysis of randomized controlled trials (RCTs) to provide evidence-based support for managing constipation.

## 2 Methods

This study was conducted in accordance with the PRISMA 2020 statement: an updated guideline for reporting systematic reviews. (Page et al., 2021). and the PRISMA for acupuncture checklist (Wang et al., 2019). The study was registered on the PROSPERO website (https://www.crd.york.ac.uk/prospero/) with the registration number: CRD42023425033.

### 2.1 Inclusion criteria

(1) Types of studies: Only RCTs with parallel group designs published in English or Chinese were included in this study. Non-RCTs, such as controlled before-and-after studies, historically controlled studies, cohort studies, and cross-sectional studies, among others, were excluded.
(2) Types of participants: Adult patients (ages 18 years or older) who meet the diagnostic criteria of functional constipation Rome II (Drossman, 1999), Rome III (Drossman, 2006), Rome Ⅳ (Drossman, 2016) or other recognized diagnostic criteria were included, regardless of gender, age and race. Pregnant and lactating women were excluded in this study.
(3) Types of interventions: Auricular acupressure, defined as the application of pressure to ear acupoints using metallic beads (or actual seeds, ceramic beads, or magnetic beads), was considered. The study included the use of auricular acupressure alone in the experimental group or in combination with the treatment in the control group. Other acupuncture therapies, such as manual acupuncture, electrical acupoint stimulation, acupoint embedding needle, and acupoint application, were excluded. There were no restrictions on the frequency and duration of the interventions.
(4) Types of comparison: Control groups included those who received basic or conventional treatment, placebo-control (sham acupressure.), and no treatment. Conventional treatment included various western medicines, no treatment consists of waiting for treatment, usual care means that control patients receive health education (no drugs involved), such as diet, exercise, emotional management, and bowel habits advice.
(5) *Types of outcome measures*:
1) *Primary outcomes*: The primary outcome measures of the study were following:
  a) Patient Assessment of Constipation-Sym ptom (PAC-SYM)
  b) Complete spontaneous bowel movements (CSBMs)
  c) Bristol Stool Form (BSF)
  d) Cleveland Clinic Score (CCS)
  e) Other indicators that may reflect improvement in spontaneous constipation.
2) *Secondary outcomes*: The secondary outcome measures of the study were as follows:
  a) Responder rate, defined as the percentage of responders among the total number of participants in each group.
  b) Cure rate.
  c) Patient Assessment of Constipation-Quality of Life (PAC-QOL) questionnaires.
  d) Adverse events related to acupressure therapy, such as tenderness or pain, dizziness, local discomfort, skin contusions, and others.

Studies were included if they report at least one of the above outcome indicators.

### 2.2 Data sources and search strategy

A comprehensive literature search was conducted from the inception of the databases until April 2023. The search was limited to articles published in Chinese and English. Eight electronic databases were searched, including four Chinese databases: China National Knowledge Infrastructure (CNKI), Wanfang, Cqvip (VIP), and Chinese Biomedical (CBM); and four English databases: PubMed, Embase, CENTRAL (Cochrane Central Register of Controlled Trials), and Web of Science. Additionally, three clinical trial registration platforms were searched: World Health Organization (WHO) International Clinical Trials Registry Platform (http://www.who.int/ictrp/en/), Chinese Clinical Trial Registry (http://www.chictr.org/en/), and ClinicalTrials.gov (https://www.clinicaltrials.gov/). The detailed retrieval process is provided in the Supplementary Material S1.

### 2.3 Study selection

**FIGURE 1:**
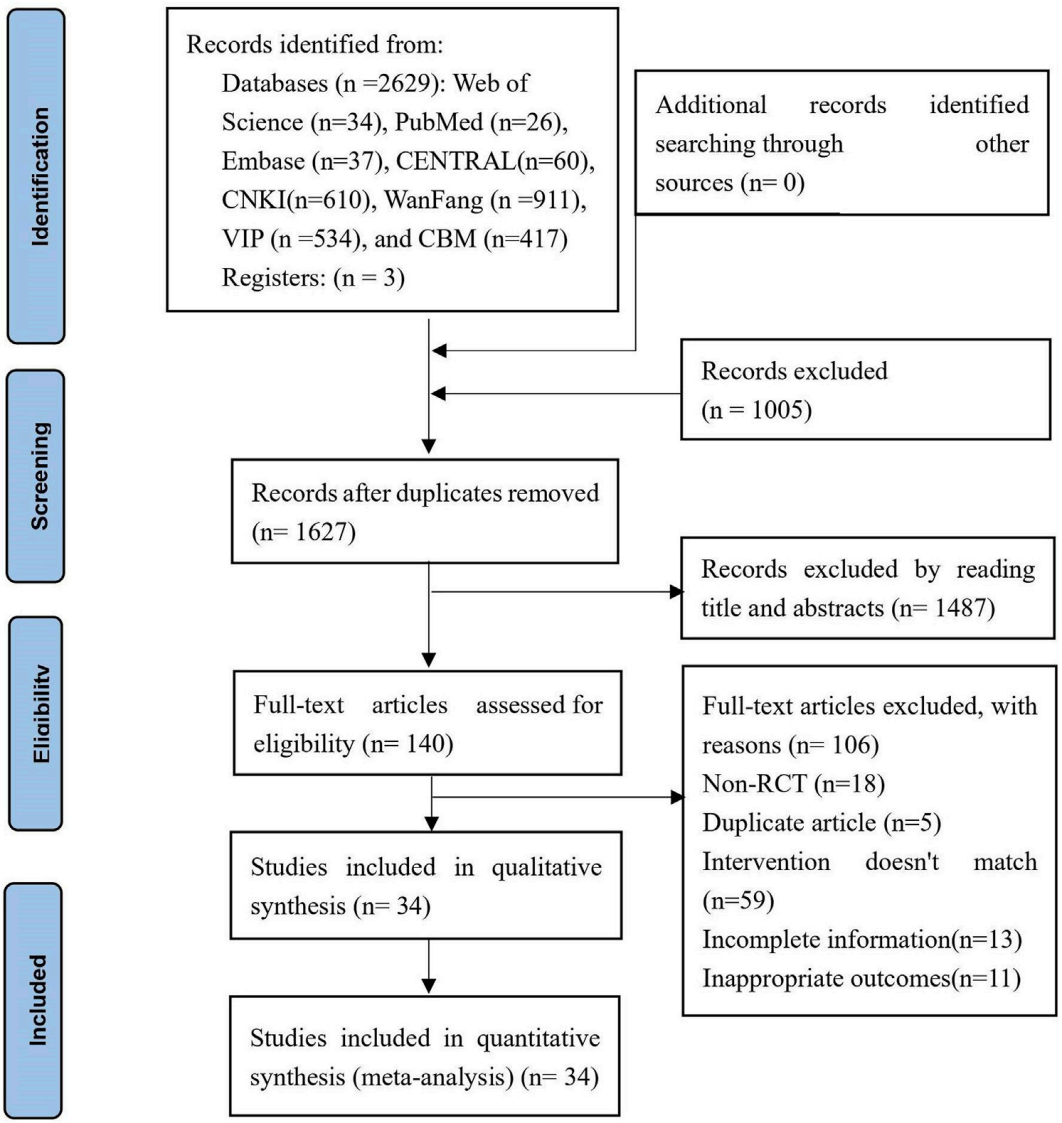
PRISMA flow diagram of the study selection process.

Using NoteExpress 3.2.0, the literature retrieved from the different databases was imported, and duplicate records were identified and removed. Two independent researchers conducted an initial screening of titles, abstracts, and other relevant information. Following the initial screening, the full texts of potentially eligible studies were obtained, and the two researchers independently assessed the full texts against the predefined inclusion and exclusion criteria. In cases of disagreement between the two researchers, a third reviewer was consulted to resolve discrepancies and make a final decision. A PRISMA flowchart illustrating the study selection process is included as Figure 1.

### 2.4 Data extraction and management

Data was independently extracted by two reviewers using a standardized form, which including the following elements: basic information (title, year of publication, first author, language, country of implementation), participant characteristics at baseline (sample size, age, gender, consistency), study design (randomization method, allocation concealment, blinding procedures, diagnostic criteria, intervention frequency and duration, acupoint composition), and outcomes. In the case of multi-arm RCTs, they were reclassified as dual-arm RCTs to ensure compatibility for result synthesis. The two reviewers conducted data extraction and cross-checking, and any discrepancies were resolved through consultation with the corresponding authors.

### 2.5 Risk of bias assessment

Two independent reviewers evaluated the quality of the included studies using the Cochrane Collaboration’s Risk of Bias Assessment tool (Cumpston et al., 2019). This assessment covered several domains, including random sequence generation (selection bias), allocation concealment (selection bias), blinding of participants and personnel (performance bias), blinding of outcome assessment (detection bias), incomplete outcome data (attrition bias), selective reporting (reporting bias), and other potential sources of bias. For instance, in the assessment of random sequence generation, the use of pseudo-random methods such as consultation dates, dates of birth, or ID numbers was considered a “high risk” of bias, while the use of true random methods such as simple random number tables or computerized random number generation was regarded as “low risk” of bias. If no specific method of randomization was stated, the risk was classified as “unclear."

### 2.6 Confidence in cumulative evidence

The Grading of Recommendations Assessment, Development, and Evaluation System (GRADE) evaluation tool (Guyatt et al., 2011) was independently used by two reviewers to assess the quality of evidence in terms of five dimensions: risk of bias, inconsistency, indirectness, imprecision, and publication bias. The quality of evidence will be classified as high, moderate, low, or very low. In the event of any disagreement, a third reviewer will be consulted to facilitate consensus.

### 2.7 Assessment of heterogeneity

Statistical heterogeneity between studies was considered acceptable when *p* ≥ 0.1 or I^2^ ≤ 50%. *p* < 0.1 or I^2^>50% means that the statistical heterogeneity is deemed significant, indicating the need for an analysis to identify the source of the heterogeneity. Sensitivity analysis or subgroup analysis was used for the assessment.

### 2.8 Data synthesis

In this study, RevMan software (version 5.4, the Nordic Cochrane Centre, Copenhagen, Denmark) was used for data synthesis and analysis. Mean difference (MD) was employed as the effect size for continuous variables, while relative risk (RR) was used for dichotomous variables. The 95% confidence interval (CI) was calculated. A significance level of *p* < 0.05 was considered statistically significant. Statistical heterogeneity was assessed using chi-squared tests and the Higgins I^2^ test. If *p* ≥ 0.1 and I^2^ ≤ 50%, indicating a high degree of homogeneity, a fixed-effects model was applied for the pooled analysis. Conversely, if *p* < 0.1 or I^2^ > 50%, indicating a significant degree of heterogeneity, a random-effects model was used. Significant clinical heterogeneity was addressed through methods such as subgroup analysis, sensitivity analysis, or a narrative summary if necessary. If the outcome data could not be pooled for meta-analysis, a narrative summary was provided instead. If there was significant heterogeneity between studies and the data were available, subgroup analyses were performed based on the varying characteristics of the included studies. If more than 10 studies were included in the analysis, the funnel plot method was used to assess publication biases. Additionally, an Egger’s test was conducted to further evaluate publication bias. The analysis for publication bias was performed using STATA software (version 16.0, Stata Corp LP, United States). A significance level of *p* < 0.05 was considered indicative of significant publication bias.

## 3 Results

### 3.1 Study selection

A total of 2,632 potentially relevant articles were identified through a systematic search across eight databases and three clinical trial registration platforms. After removing 1,005 duplicates, a preliminary screening of titles and abstracts resulted in the exclusion of 1,487 studies. From the remaining 140 studies, 106 were excluded after reading the full text. Ultimately, 34 RCTs (Zhong, 2007; Zhang, 2009; Liu et al., 2011; Chen et al., 2012; Qian, 2012; Xu et al., 2013; Yang and Fu, 2013; Hu et al., 2014; Li et al., 2014; Yang et al., 2014; Ye and He, 2014; Ji et al., 2015; Shen et al., 2015; Chen and Hou, 2016; Huang, 2016; Ji et al., 2016; Qu and Ye, 2016; Liu and Jiang, 2017; Du and Xu, 2018; Li et al., 2018; Liu, 2018; Shin and Park, 2018; Wang, 2019; Xu, 2019; Jung and Chang, 2020; Wang et al., 2020; Chen et al., 2021; Fu et al., 2021; Jie et al., 2021; Wu et al., 2021; Wang et al., 2022; Zhao and Wang, 2022; Aminizadeh et al., 2023; Xu, 2023) met the inclusion criteria, with sample sizes ranging from 25 to 50 participants, involving a total of 2,465 patients. The screening process is presented in Figure 1, illustrating the flow diagram.

### 3.2 Study characteristics

The main characteristics of the included RCTs are showed in Table 1. The studies, conducted between 2007 and 2023, were predominantly carried out in China, with one study conducted in Hong Kong (Li et al., 2014)), Iran (Aminizadeh et al., 2023), and the Republic of Korea (Shin and Park, 2018; Jung and Chang, 2020). Thirty-two studies were published in Chinese (Zhong, 2007; Zhang, 2009; Liu et al., 2011; Chen et al., 2012; Qian, 2012; Xu et al., 2013; Yang and Fu, 2013; Hu et al., 2014; Yang et al., 2014; Ye and He, 2014; Ji et al., 2015; Shen et al., 2015; Chen and Hou, 2016; Huang, 2016; Ji et al., 2016; Qu and Ye, 2016; Liu and Jiang, 2017; Du and Xu, 2018; Li et al., 2018; Liu, 2018; Wang, 2019; Xu, 2019; Wang et al., 2020; Chen et al., 2021; Fu et al., 2021; Jie et al., 2021; Wu et al., 2021; Wang et al., 2022; Zhao and Wang, 2022; Xu, 2023) while four were published in English (Li et al., 2014; Shin and Park, 2018; Jung and Chang, 2020; Aminizadeh et al., 2023). Among the included studies, 34 were two-arm studies (Zhong, 2007; Zhang, 2009; Liu et al., 2011; Chen et al., 2012; Qian, 2012; Xu et al., 2013; Yang and Fu, 2013; Hu et al., 2014; Yang et al., 2014; Ye and He, 2014; Ji et al., 2015; Shen et al., 2015; Chen and Hou, 2016; Huang, 2016; Ji et al., 2016; Qu and Ye, 2016; Liu and Jiang, 2017; Du and Xu, 2018; Li et al., 2018; Liu, 2018; Shin and Park, 2018; Wang, 2019; Xu, 2019; Jung and Chang, 2020; Wang et al., 2020; Chen et al., 2021; Fu et al., 2021; Jie et al., 2021; Wu et al., 2021; Wang et al., 2022; Zhao and Wang, 2022; Aminizadeh et al., 2023; Xu, 2023), and one was a three-arm study (Li et al., 2014). The age range of patients with constipation was 18 years and older, with seven studies specifically including elderly patients (Zhang, 2009; Yang and Fu, 2013; Ye and He, 2014; Chen and Hou, 2016; Fu et al., 2021; Wang et al., 2022; Aminizadeh et al., 2023). Some studies included patients with additional conditions, such as subarachnoid hemorrhage (Qian, 2012), post-fracture (Yang and Fu, 2013; Liu and Jiang, 2017), stroke (Xu et al., 2013; Ji et al., 2015; Ji et al., 2016), post-craniotomy (Wu et al., 2021), opioid use (Yang et al., 2014), morphine use (Qu and Ye, 2016), diabetes (Hu et al., 2014), schizophrenia (Ye and He, 2014; Jie et al., 2021; Xu, 2023), hemodialysis (Shen et al., 2015; Jung and Chang, 2020; Fu et al., 2021), heart failure (Li et al., 2018), hypertension (Chen and Hou, 2016), and breast cancer patients receiving chemotherapy (Shin and Park, 2018). The treatment duration ranges from 1 week to 8 weeks. Adverse reactions were reported in seven studies (Zhong, 2007; Yang et al., 2014; Liu, 2018; Fu et al., 2021; Zhao and Wang, 2022; Aminizadeh et al., 2023; Xu, 2023), while two studies (Zhong, 2007; Xu, 2023) specifically mentioned that AA had no adverse reactions. The four most commonly selected auricular acupoints in the included studies were the Large intestine (n = 33), Spleen (n = 27), San Jiao (n = 26), and Rectum (n = 23).

**TABLE 1:** Basic characteristics of the included studies.

References	Comorbid conditions	Sample (T/C)	Age		Interventions		Acupoints	Dose or frequency	Duration	Outcomes	Safety
			T	C	T	C					
Zhong, (2007)	NO	30/30	45.13 ± 15.51	45.90 ± 16.03	AA	CT	Main acupoint: Lung, Spleen, Large Intestine, Rectum, Subcortex, and Constipation	Press 3–5 times daily each point	20d	Responder rate, Cure rate	R
							Matching acupoint: Stomach, Abdomen, and San Jiao				
Zhang, (2009)	NO	30/30	NR	NR	AA	CT	Main acupoint:Rectum, Large Intestine, Constipation, Subcortex, and Lung	Press 3–5 times daily each point, for 5 min each time. Stimulate to the maximum tolerance level	8w	Responder rat**,** Cure rate	NR
							Matching acupoint				
							Excess syndrome: Stomach, San Jiao, and Abdomen				
							Deficiency syndrome: Spleen, and Kidney				
Liu, (2011)	NO	40/40	NR	NR	AA	UC	Main acupoint:Rectum, Large Intestine, Constipation, Sympathetic, San Jiao, and Subcortex. (2 acupoints per session.)	Press 3–4 times daily each point	18d	Responder rate	NR
							Matching acupoint: Liver, Spleen, Stomach, Lung, and Endocrine. (2–3 acupoints per session.)				
Chen et al. (2012)	NO	35/35	79.6 ± 6.5	79.2 ± 5.8	AA	UC	Large Intestine, San Jiao, Lung, Spleen, and Stomach	Press 3 times daily each point, for 3 min each time	40d	Responder rate**,** Cure rate	NR
Qian, (2012)	Subarachnoid hemorrhage	42/42	37.1	37.1	AA	UC	Main acupoint:Large Intestine, San Jiao, Spleen, Abdomen, Digestive system, and Subcortex	Press 2–3 times daily each point, for 3–5 min each time	2w	Responder rate**,** Cure rate	NR
							Matching acupoint:Lung, Sigmoid colon				
Xu, (2013)	Stroke	40/39	58.48 ± 9.01	60.46 ± 7.34	AA	CT	Main acupoint: Large Intestine, San Jiao**,** Constipation, Spleen, Digestive system, and Subcortex	Press 3–4 times daily each point, 20s/one point	30d	Responder rate**,** Cure rate	NR
							Matching acupoint: Ear apex, Adrenal gland or Liver**,** Sympathetic or Kidney**,** Adrenal gland or Lung**,** Shenmen or Heart**,** Small Intestine or Rectum**,** Kidney or Kidney				
Yang et al, (2013)	Post-fracture	43/43	NR	NR	AA	CT	Large Intestine, Rectum, Constipation, and Subcortex	Press 3–4 times daily each point, 1–2 min/one point	NR	Responder rate	NR
Yang et al, (2014)	Opioids use	30/30	63.63 ± 13.46	63.23 ± 11.45	AA+ CT	CT	Subcortex, Brainstem, Abdomen, and Large Intestine	Press 4 times daily each point, 2 min/one point	2w	BFI, PAC-QOL	R
							Excess syndrome: Liver, Spleen, and San Jiao				
							Deficiency syndrome: Lung, Kidney, and Spleen				
Hu et al, (2014)	Diabetes	30/30	58.94 ± 14.14	60.16 ± 11.29	AA+ UC	UC	Excess syndrome: Rectum, Large Intestine, Lung, San Jiao, and Constipation	Excess syndrome	2w	Responder rate**,** Cure rate	NR
							Deficiency syndrome: Rectum, Large Intestine, Lung, San Jiao, Constipation, and Spleen	Press 5–6 times daily each point, for 3–5 min each time. Deficiency syndrome: Press 3–4 times daily each point, for 1–3 min each time			
Ye and He, (2014)	Schizophrenia	50/50	63.7 ± 9.6	64.2±	AA+ UC	UC	Large Intestine, Constipation, Spleen, and Rectum	Press 3 times daily each point, for 3 minutes each time	40d	Responder rate**,** Cure rate	NR
				10.3							
Ji et al, (2015)	Stroke	40/40	61. 3 ±	59. 6 ± 9. 7	AA+ CT	CT	Rectum, Large Intestine, Lung, San Jiao, Sympathetic, and Constipation	Press 3 times daily each point, 3 min/one point	8d	Responder rate**,** Cure rate	NR
			8. 6								
Shen et al, (2015)	Hemodialysis	45/45	NR	NR	AA+ CT	CT	Main acupoint: Large Intestine, Small Intestine, and Rectum	Press 3–5 times daily each point, 3 min/one point	30d	Responder rate**,** Cure rate	NR
							Matching acupoint:Spleen, Kidney, and Endocrine				
Chen and Hou, (2016)	Hypertension	32/32	NR	NR	AA+ UC	UC	Main acupoint:Rectum**,** Large Intestine, Spleen, and Constipation	NR	4w	Responder rate**,** Cure rate	NR
							Matching acupoint:San Jiao, Small Intestine, and Stomach				
Huang, (2016)	NR	50/50	40.6 ± 8.9	41.6±	AA	CT	Large Intestine, Rectum, Constipation, San Jiao, and Lung (3–4 acupoints per session.)	Press 3–5 times daily each point, for 1–2 min each time	1w	Responder rate**,** Cure rate	NR
				7.7							
Ji et al, (2016)	Stroke	45/45	61 ± 9	60 ± 10	AA+ UC	UC	Rectum, Large Intestine, Constipation, Lung, San Jiao, and Sympathetic	Press 3 times daily each point, for 3 minutes each time	8d	Responder rate**,** Cure rate	NR
Qu and Ye, (2016)	Morphine use	50/50	NR	NR	AA+ UC	CT + UC	San Jiao, Spleen, Stomach, Liver, Lung, and Large Intestine	Press 5–6 times daily each point		Responder rate**,** Cure rate	NR
Liu and Jiang, (2017)	Post-fracture	30/30	49.7 ± 2.6	48.6±	AA+ UC	Sham AA+ UC+	Spleen, Small Intestine, Large Intestine, Kidney, and Sympathetic	Press 5 times daily each point, 1 min/one point	1w	Responder rate	NR
				2.5							
Du and Xu, (2018)	NR	30/30	NR	NR	AA	CT	Main acupoint: Lung, Spleen, Large Intestine, Rectum, Subcortex, and Constipation	Press 3–5 times daily each point, gently rotate each acupoint 27 times	2w	Responder rat**,** Cure rate	NR
							Matching acupoint: Stomach, Abdomen, and San Jiao				
Li et al, (2018)	Heart failure	25/25	NR	NR	AA+ UC	UC	Main acupoint: Large Intestine, Small Intestine, Rectum, Shenmen, Lung, and Stomach. (5 acupoints per session.)	Press 3 times daily each point, 3 min/one point	1w	Responder rate, Cure rate	NR
							Matching acupoint: Spleen, Kidney, Endocrine, and San Jiao. (2 acupoints per session.)				
Liu, (2018)	NR	32/33	50.16 ± 13.58	50.13 ± 11.94	AA	CT	Large Intestine, Small Intestine, Rectum, Liver, Spleen, and Lung	Press 5 times daily each point	2w	Responder rate, Cure rate, BSF, PAC-QOL, CSBMs	R
Wang et al, (2019)	Alzheimer’s Disease	30/30	72.3±	71.1±	AA+ UC	UC	Constipation, Lung, Large Intestine, Kidney, Spleen, and San Jiao	Press 4–5 times daily each point, 1–2 min/one point	2w	Responder rate, Cure rate	NR
			415	3.58							
Xu, (2019)	NR	30/30	49.77 ± 7.81	48.87 ± 10.01	AA+ CT	CT	Main acupoint: Lung**,** Spleen**,** Large Intestine**,** Rectum**,** Subcortex, and Constipation	Press 3–5 times daily each point	20d	PAC-QOL, Responder rate, Cure rate	NR
							Matching acupoint:Stomach**,** Abdomen, and San Jiao				
Wang et al. (2020)	NR	30/30	NR	NR	AA+ CT+	CT	Heart, Shenmen, Sympathetic, Large Intestine, and Rectum	Press 3 times daily each point, 1min/one point	24d	Responder rate, Cure rate	NR
Chen et al. (2021)	NR	45/45	21.56 ± 1.89	21.00 ± 2.27	AA+ UC	UC	Shenmen, Sympathetic, Large Intestine, Small Intestine, Stomach, and San Jiao	Press 4 times daily each point, 30s/one point	2w	PAC-QOL	NR
Fu et al. (2021)	Hemodialysis	25/25	69.73 ± 3.52	69.54 ± 3.6	AA+ UC	UC	Small Intestine, Sigmoid colon, Rectum, Kidney, Bladder, Ureter, Duodenum, Appendix, and Large Intestine	Press 3–5 times daily each point, 1–2 min/one point	4w	NR	R
Jie et al, (2021)	Schizophrenia	28/29	55.82 ± 7. 35	53.40 ± 8. 70	AA	Sham AA	Liver, Spleen	Self-apply pressure multiple times daily, until a local sensation of soreness, numbness, swelling, or pain is felt	8w	BSF, PAC- SYM	NR
Wu et al., 2021	post-craniotomy	49/49	46.37 ± 3.54	46.33 ± 3.50	AA+ CT	CT	Spleen, Stomach, Kidney, Small Intestine, Large Intestine, Rectum, Constipation, and Subcortex	Press 3–5 times daily each point	4w	PAC-QOL, Responder rate, Cure rate	NR
Wang et al., 2022	NR	40/40	73.8 ± 7.1	75.1 ± 7.6	AA+ UC	UC	Main acupoint: Constipation, Large Intestine, and San Jiao	Press 3–5 times daily each point, 1min/one point	60d	Responder rate, Cure rate, BSF, CSBMs	NR
							Matching acupoint:Lung, Spleen, and Sympathetic (1–2 acupoints per session)				
Zhao and Wang. (2022)	Stroke	49/49	53.08 ± 4.18	53.05 ± 4.20	AA+ UC	UC	Main acupoint: Rectum, Small Intestine, Large Intestine, Sympathetic, and Subcorte	Press 5 times daily each point. 1–2min/one point	10d	Responder rate, BSF, PAC-QOL	R
							Matching acupoint: Stomach, Spleen, Brain, Heart or San Jiao, Kidney, Liver, Endocrine or Spleen, Lung, Kidney, and Liver				
Xu et al, (2023)	Schizophrenia	25/25	32.37 ± 8.82	32.83±	AA+ CT	CT	Constipation, Large Intestine, Small Intestine, Spleen, Stomach, and San Jiao	Press 25 times daily	4w	CCS, Responder rate, Cure rate	R
				8.92							
Aminizadeh et al., 2023	NR	27/26	74.20 ± 5.37	72.77 ± 4.20	AA	Sham AA	Large Intestine, Rectum, San Jiao, spleen, Lung, Sympathetic, and subcortex	Press 3–4 times daily	10d	PAC-QOL, PAC-SYM	R
Jung and Chang. (2020)	Hemodialysis	30/30	67.30 ± 10.60	61.50 ± 1.31	AA	Sham AA	Large Intestine, San Jiao, Middle triangular fossa, Spleen, and Upper tragus	Force of finger pressure as 0.3–0.5 kg	4w	CAS	NR
Li et al, (2014)	NR	33/33/33	84.2 ± 7.29	83.8 ± 7.19/86.9 ± 8.01	AA/	AA/Sham AA	Large Intestine, Rectum, San Jiao, Spleen, Lung, Sympathetic, and Subcortex	No pressure	10d	PAC—SYM	NR
										PAC—QOL	
Shin and Park, (2018)	Breast Cancer	26/26	NR	NR	AA	UC	Intestine, Rectum, San Jiao, Spleen, Lung, Sympathetic, and subcortex	Press 3–4 times daily	6w	CAS, BSF, PAC-QOL	NR

T, test group; C, control group; AA, auricular acupressure; UC, usual care; CT, conventional treatment; w, week; d, day; R: report; NR, not report; PAC-SYM, Patient Assessment of Constipation-Symptom; CSBMs, Complete spontaneous bowel movements; BSF, bristol stool form; CAS, constipation assessment scale; PAC-QOL, Patient Assessment of Constipation-Quality of Life questionnaires; CCS, cleveland clinic score; BFI: bowel functional index.

### 3.3 Risk of bias

**FIGURE 2:**
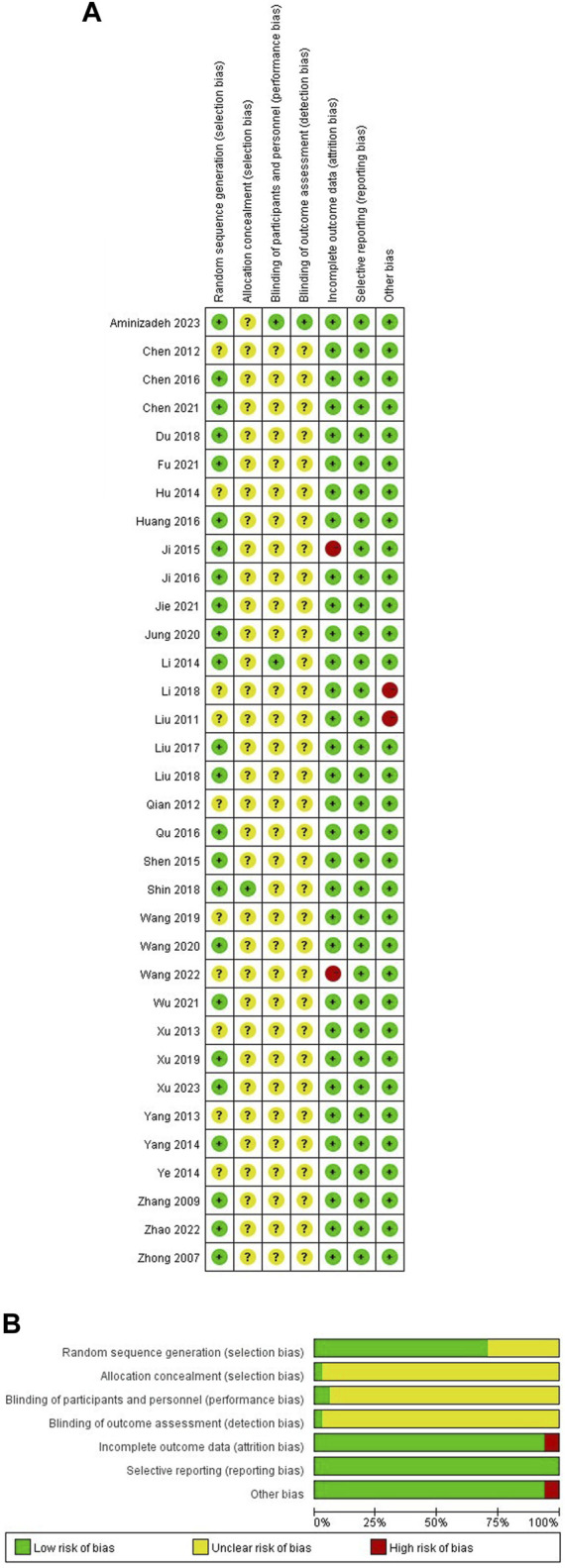
Risk of bias of RCTs **(A)** Risk of bias summary; **(B)** Risk of bias graph.

All the included studies mentioned the term “randomized” or “random” in their methodology. One of the RCTs (Shin and Park, 2018)included in the study implemented concealed allocation and received a “low risk” rating for allocation concealment. The majority of the included RCTs received an “unclear” rating for blinding of participants and personnel, with the exception of two studies (Li et al., 2014; Aminizadeh et al., 2023) that utilized a sham AA procedure. One (Li et al., 2014) of the RCTs included in the study implemented blinding of outcome assessment and received a “low risk”. Two studies (Ji et al., 2015; Wang et al., 2022) were classified as “high risk” due to without providing a description of the reasons for dropout. In terms of other biases, four studies were classified as high risk. Among them, two studies (Liu et al., 2011; Li et al., 2018) did not report baseline consistency, and two studies (Yang and Fu, 2013; Qu and Ye, 2016) did not report treatment duration. The detailed results are depicted in Figure 2.

### 3.4 Meta-analysis

The summary of the meta-analysis results is provided in Table 2, with a detailed explanation below.

**TABLE 2:** Summary of meta-analysis results.

Outcomes		Research type	Country	Number	T/C	Heterogeneity test results		Effect model (F/R)	Meta-analysis results	
						*p*-value	I^2^		MD/RR and 95%CI	*p*-value
PAC-SYM										
Treatment(s)	AA vs. sham AA	RCT	China and Iran	2	58/59	*p* = 0.52	I^2^ = 0%	R	MD = -3.04, 95% CI: [-0.49, −0.19]	*p* < 0.0001
	AA vs. UC	RCT	Hong Kong (China)	2	54/54	*p* = 0.20	I^2^ = 38%	R	MD = -0.04, 95%CI: [-0.24, 0.15]	*p* = 0.68
Age (years)	≥60	RCT	Hong Kong (China) and Iran	3	84/84	*p* = 0.01	I^2^ = 77%	R	MD = -0.15, 95%CI: [-0.38, 0.09]	*p* = 0.21
	≥18	RCT	China	1	28/28	-	-	-	MD = -1.89, 95%CI: [-6.61, 2.83]	*p* = 0.43
CSBMs		RCT	China	2	69/69	*p* = 0.93	I^2^ = 0%	F	MD = 1.22, 95% CI: [0.68, 1.77]	*p* < 0.0001
Age (years)	≥60	RCT	China	1	38/38	-	-	-	MD = 1.20, 95% CI: [0.45, 1.95]	*p* = 0.002
	≥18	RCT	China	1	31/31	-	-	-	MD = 1.25, 95% CI: [0.45, 2,05]	*p* = 0.002
BSF		RCT	China and Republic of Korea	4	123/124	*p* = 0.0008	I^2^ = 82%	R	MD = 0.72, 95% CI: [0.15, 1.28]	*p* = 0.01
Age (years)	≥60	RCT	China	1	38/38	-	-	-	MD = 0.70, 95% CI: [0.26, 1.14]	*p* = 0.002
	≥18	RCT	China and Republic of Korea	3	85/86	*p* = 0.0005	I^2^ = 87%	R	MD = 0.75, 95% CI: [-0.08, 1.59]	*p* = 0.08
CAS		RCT	Republic of Korea	2	52/53	*p* = 0.02	I^2^ = 80%	R	MD = -3.28, 95%CI: [-5.95, −0.60]	*p* = 0.02
Responder rate										
Treatment(s)	AA vs. CT	RCT	China	7	254/253	*p* = 0.13	I^2^ = 39	R	RR = 1.24, 95%CI: [1.11,1.39]	*p* = 0.0002
	AA plus CT vs. CT	RCT	China	6	219/219	*p* = 0.002	I^2^ = 74%	R	RR = 1.23, 95%CI: [1.04, 1.46]	*p* = 0.02
	AA vs. UC	RCT	China	3	117/115	*p* = 0.01	I^2^ = 78%	R	RR = 1.28, 95%CI: [0.99, 1.66]	*p* = 0.06
	AA+ UC vs. UC	RCT	China	8	294/294	*p* < 0.00001	I^2^ = 92%	R	RR = 1.34, 95%CI: [1.05, 1.70]	*p* = 0.02
Age (years)	18–60	RCT	China	5	195/195	*p* = 0.57	I^2^ = 0%	F	RR = 1.21, 95%CI: [1.11, 1.31]	*p* < 0.0001
	≥60	RCT	China	7	258/256	*p* < 0.00001	I^2^ = 88%	R	RR = 1.21, 95%CI: [1.01, 1.46]	*p* = 0.04
	≥18	RCT	China	14	511/510	*p* < 0.00001	I^2^ = 73%	R	RR = 1.33, 95%CI: [1.17, 1.52]	*p* < 0.0001
Cure rate										
Treatment(s)	AA vs. CT	RCT	China	6	211/210	*p* = 0.87	I^2^ = 0%	F	RR = 2.68, 95% CI [1.68, 4.28]	*p* < 0.0001
	AA+ UC vs. UC	RCT	China	7	245/245	*p* = 0.54	I^2^ = 0%	F	RR = 1.79, 95% CI [1.34, 2.38]	*p* < 0.0001
	AA vs. UC	RCT	China	2	77/75	*p* = 0.90	I^2^ = 0%	F	RR = 1.84, 95% CI [1.30, 2.62]	*p* = 0.0007
	AA plus CT vs. CT	RCT	China	6	219/219	*p* = 0.87	I^2^ = 0%	F	RR = 1.59, 95% CI [1.21, 2.09]	*p* = 0.0008
Age (years)	18–60	RCT	China	3	116/116	*p* = 0.38	I^2^ = 0%	F	RR = 0.22, 95% CI [0.10, 0.34]	*p* = 0.0003
	≥60	RCT	China	6	215/213	*p* = 0.59	I^2^ = 0%	F	RR = 0.19, 95% CI [0.11, 0.27]	*p* < 0.00001
	≥18	RCT	China	13	471/470	*p* = 0.79	I^2^ = 0%	F	RR = 0.13, 95% CI [0.08, 0.18]	*p* < 0.00001
PAC-QOL										
Treatment(s)	AA+ UC vs. UC	RCT	China	2	94/94	*p* = 0.75	I^2^ = 0%	R	MD = −4.81, 95%CI: [-5.55, −4.08]	*p* < 0.00001
	AA vs. UC	RCT	Hong Kong (China) and Republic of Korea	3	80/80	*p* < 0.0001	I^2^ = 91%	R	MD = −0.28, 95% CI: [-0.70, 0.14]	*p* = 0.19
	AA plus CT vs. CT	RCT	China	3	109/109	*p* < 0.00001	I^2^ = 99%	R	MD = −11.21, 95% CI: [-24.12, 1.70]	*p* = 0.09
Age (years)	18–60	RCT	China	3	143/143	*p* < 0.00001	I^2^ = 97%	R	MD = −8.97, 95% CI: [-15.81, −2.13]	*p* = 0.01
	≥60	RCT	Iran and Hong Kong (China)	3	84/84	*p* < 0.0001	I^2^ = 89%	R	MD = −0.17, 95% CI: [-0.49, 0.14]	*p* = 0.27
	≥18	RCT	China and Republic of Korea	4	117/117	*p* < 0.00001	I^2^ = 98%	R	MD = −5.90, 95% CI: [-7.87, −3.93]	*p* < 0.00001

MD, weighted mean difference; RR, risk ratio; CI, confidence interval; T, test group; C, control group; F, fixed effects model; R, random effects model; PAC-SYM, Patient Assessment of Constipation-Symptom; CSBMs, Complete spontaneous bowel movements; BSF, bristol stool form; CAS, constipation assessment scale; PAC-QOL, Patient Assessment of Constipation-Quality of Life questionnaires; AA, auricular acupressure; UC, usual care; CT, conventional treatment; RCT, randomized controlled trial.

#### 3.4.1 Primary outcome

##### 3.4.1.1 PAC-SYM

**FIGURE 3:**
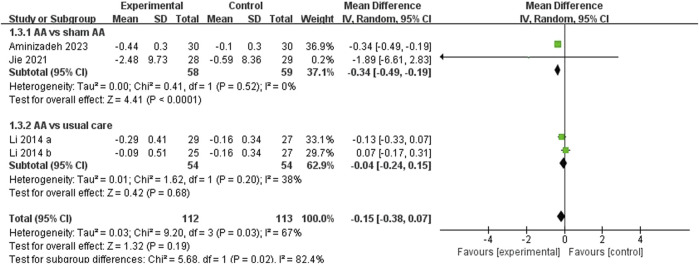
Meta-analysis and forest plot for PAC-SYM.

Among the included RCTs, three studies (Li et al., 2014; Jie et al., 2021; Aminizadeh et al., 2023) reported PAC-SYM as an outcome measure, with one of them (Li et al., 2014) being a three-arm study. The pooled data analysis revealed that there was no significant decrease in PAC-SYM overall (MD = −0.15, 95% CI: [-0.38, 0.07], *p* = 0.19, I^2^ = 67%) (Figure 3). A subgroup analysis was conducted based on different comparators, and the results indicated that Auricular acupressure (AA) was more effective than sham AA (MD = −0.34, 95% CI: [-0.49, −0.19], *p* < 0.0001, I^2^ = 0%). However, no significant difference was observed when comparing AA to usual care (MD = −0.04, 95% CI: [-0.24, 0.15], *p* = 0.68, I^2^ = 38%). When subgroup analysis based on patient age was conducted, no statistically significant differences in PAC-SYM improvement were observed across all age groups with AA (Table 2).

##### 3.4.1.2 CSBMs

**FIGURE 4:**
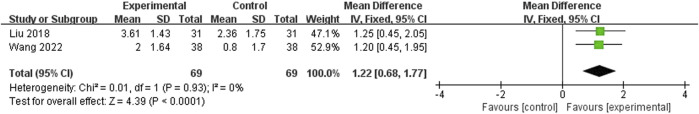
Meta-analysis and forest plot for CSBMs.

Among the included RCTs, two studies (Liu, 2018; Wang et al., 2022) reported CSBMs as an outcome, the pooled data analysis indicated that AA could increase CSBMs [MD = 1.22, 95% CI (0.68, 1.77), *p* < 0.0001, I^2^ = 0%] (Figure 4).

##### 3.4.1.3 BSF

**FIGURE 5:**
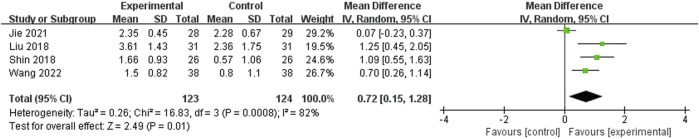
Meta-analysis and forest plot for BSF.

**FIGURE 6:**
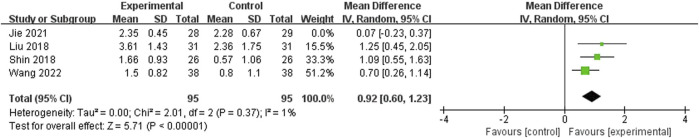
Meta-analysis and forest plot for BSF (a study has been excluded).

Five studies (Liu, 2018; Shin and Park, 2018; Jie et al., 2021; Wang et al., 2022; Zhao and Wang, 2022) reported BSF as an outcome, however, one study (Zhao and Wang, 2022) with a different scoring rule was excluded from the meta-analysis. The pooled data indicated a significant improvement in BSF (MD = 0.72, 95%CI: [0.15,1.28], *p* = 0.01, I^2^ = 82%) (Figure 5). Given to the high heterogeneity, a sensitivity analysis was conducted. When one study (Jie et al., 2021) was removed, the heterogeneity decreased (MD = 0.92, 95%CI: [0.60, 1.23], *p* < 0.00001, I^2^ = 1%) (Figure 6).

##### 3.4.1.4 Constipation Assessment Scale (CAS)

**FIGURE 7:**
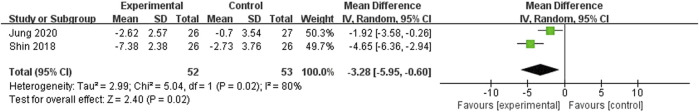
Meta-analysis and forest plot for CAS.

CAS was measured after AA treatment in two studies (Shin and Park, 2018; Jung and Chang, 2020), due to the high heterogeneity (I^2^ = 80%), an analysis was conducted using a random-effects model. The meta-analysis indicated a significant decrease in CAS (MD = -3.28, 95%CI: [-5.95, −0.60], *p* = 0.02, I^2^ = 80%) (Figure 7).

#### 3.4.2 Secondary outcomes

##### 3.4.2.1 Responder rate

**FIGURE 8:**
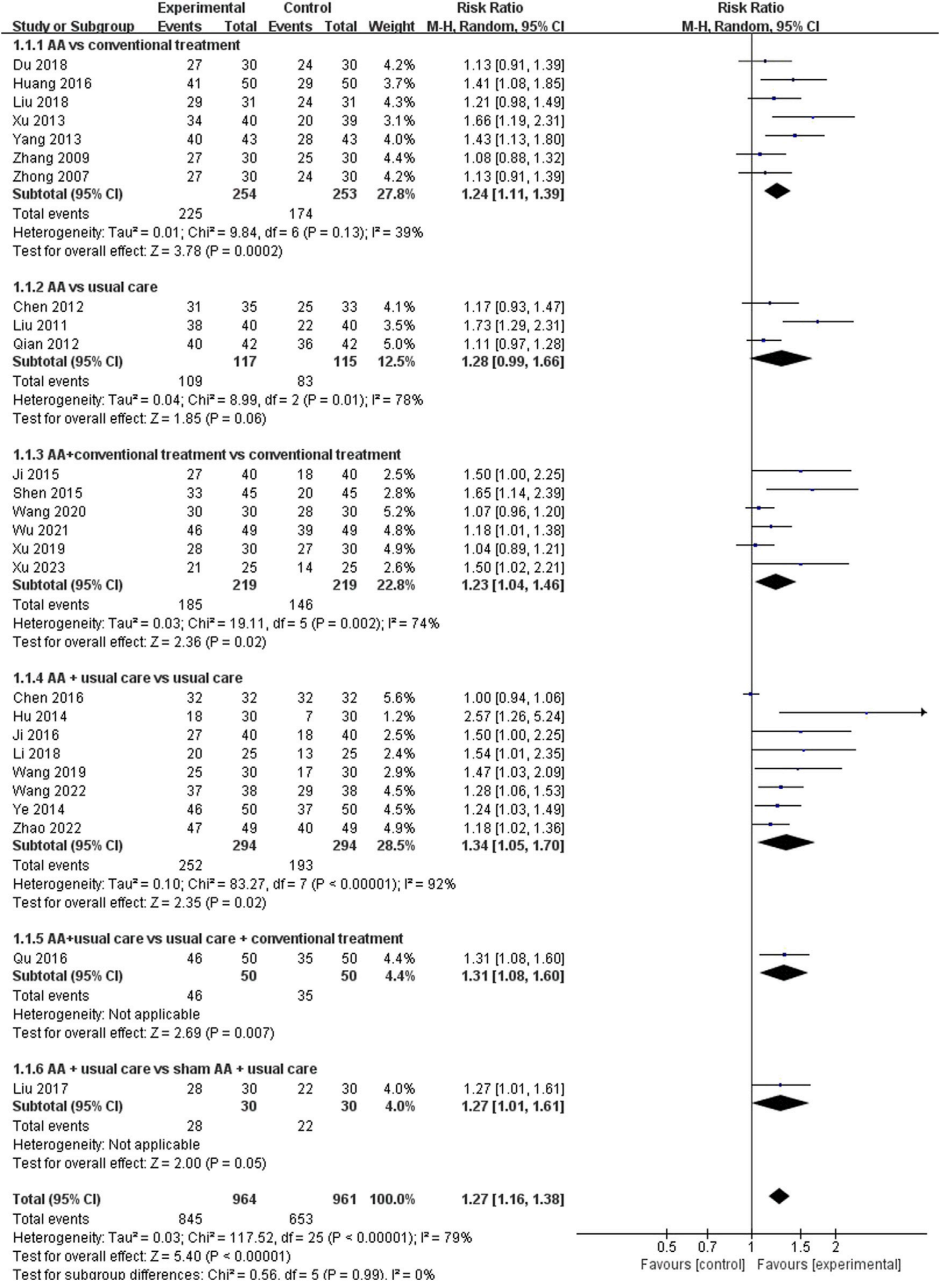
Meta-analysis and forest plot for responder rate.

Twenty-six studies (Zhong, 2007; Zhang, 2009; Liu et al., 2011; Chen et al., 2012; Qian, 2012; Xu et al., 2013; Yang and Fu, 2013; Hu et al., 2014; Ye and He, 2014; Ji et al., 2015; Shen et al., 2015; Chen and Hou, 2016; Huang, 2016; Ji et al., 2016; Qu and Ye, 2016; Liu and Jiang, 2017; Du and Xu, 2018; Li et al., 2018; Liu, 2018; Wang, 2019; Xu, 2019; Wang et al., 2020; Wu et al., 2021; Wang et al., 2022; Zhao and Wang, 2022; Xu, 2023) reported the responder rate. The pooled results indicated that overall, there was a significantly higher responder rate in the experimental group compared to the control group (RR = 1.27, 95%CI: [1.16, 1.38], *p* < 0.00001, I^2^ = 79%). A subgroup analysis was performed based on different comparators. The results suggested that AA alone (RR = 1.24, 95%CI: [1.11, 1.39], *p* = 0.0002, I^2^ = 39%) or plus conventional treatment (RR = 1.23, 95%CI: [1.04, 1.46], *p* = 0.02, I^2^ = 74%) was superior to conventional treatment. AA combined with usual care was superior to usual care (RR = 1.34, 95%CI: [1.05, 1.70], *p* = 0.02, I^2^ = 92%), and removing the article by Chen et al. (Chen and Hou, 2016) reduced the heterogeneity (RR = 1.32, 95%CI: [1.16, 1.49], *p* < 0.0001, I^2^ = 36%). Similarly, the combination of AA with usual care demonstrated superior outcomes compared to combining usual care with conventional treatment (RR = 1.31, 95%CI: [1.08, 1.60], *p* = 0.007, I^2^ = not applicable). However, no significant difference in responder rate was observed when AA compared to usual care (RR = 1.28, 95%CI: [0.99, 1.66], *p* = 0.06, I^2^ = 78%) or combined with usual care compared to usual care plus sham AA (RR = 1.27, 95%CI: [1.01, 1.61], *p* = 0.05, I^2^ = not applicable) (Figure 8). In addition, when subgroup analysis based on patient age was performed, AA showed statistically significant differences in improving responder rate among different age groups (Table 2).

##### 3.4.2.2 Cure rate

**FIGURE 9:**
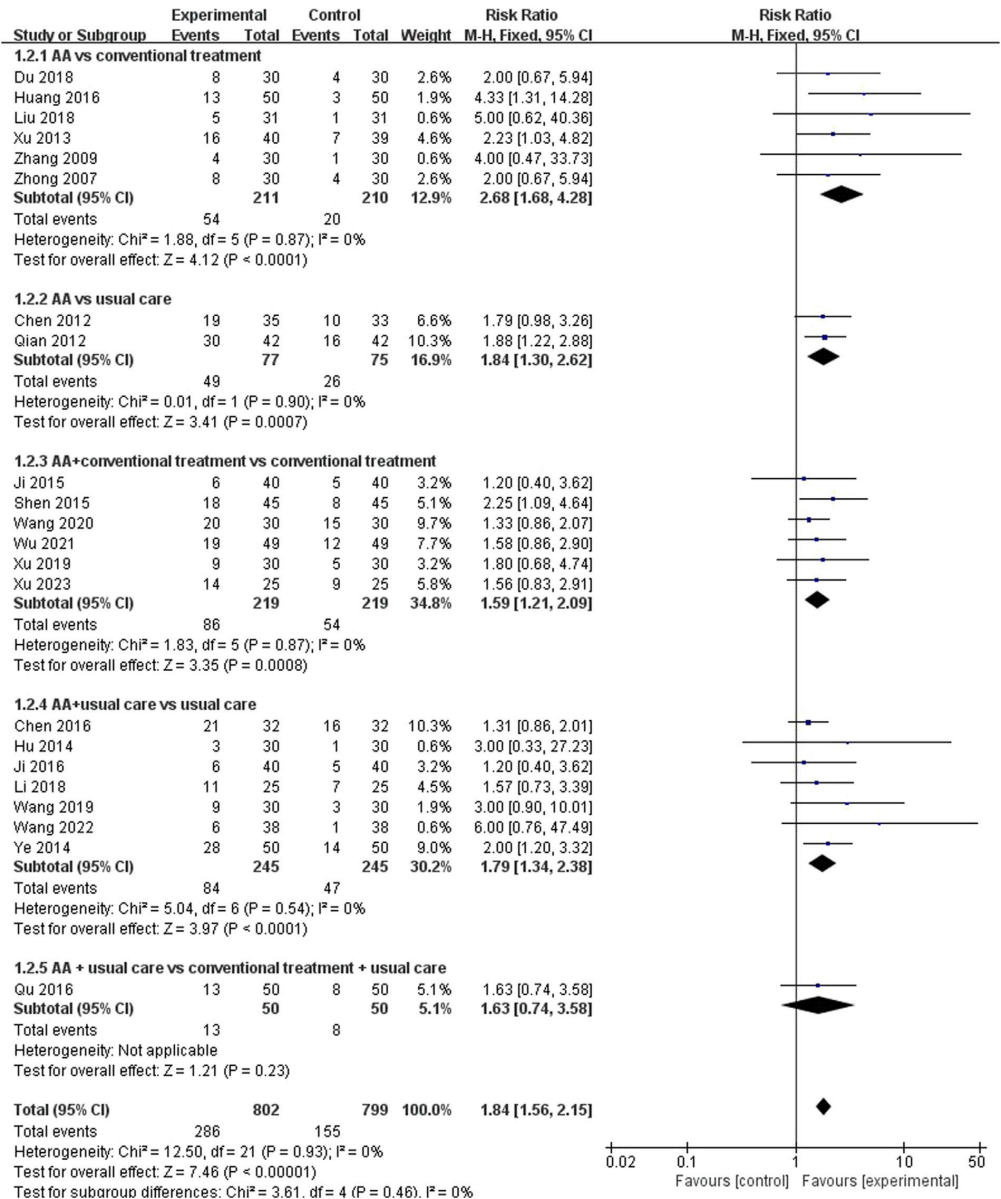
Meta-analysis and forest plot for cure rate.

There are twenty-two studies (Zhong, 2007; Zhang, 2009; Chen et al., 2012; Qian, 2012; Xu et al., 2013; Hu et al., 2014; Ye and He, 2014; Ji et al., 2015; Shen et al., 2015; Chen and Hou, 2016; Huang, 2016; Ji et al., 2016; Qu and Ye, 2016; Du and Xu, 2018; Li et al., 2018; Liu, 2018; Wang, 2019; Xu, 2019; Wang et al., 2020; Wu et al., 2021; Wang et al., 2022; Xu, 2023) reported the cure rate as an outcome. The fixed-effect model was employed based on the results of heterogeneity test (*p* = 0.93, I^2^ = 0%). Meta-analysis showed that the treatment group can significantly increase cure rate among patients with constipation (RR = 1.84, 95% CI [1.56, 2.15], *p* < 0.00001). Subgroup analysis was performed according to different comparators, indicating that AA alone (RR = 2.68, 95% CI [1.68, 4.28], *p* < 0.0001, I^2^ = 0%) or combined with conventional treatment (RR = 1.59, 95% CI [1.21, 2.09], *p* = 0.0008, I^2^ = 0%) was superior to conventional treatment. Similarly, AA alone (RR = 1.84, 95% CI [1.30, 2.62], *p* = 0.0007, I^2^ = 0%) or combined with usual care (RR = 1.79, 95% CI [1.34, 2.38], *p* < 0.0001, I^2^ = 0%) could increase the cure rate when compared with usual care, however, there was no significant difference was observed when AA combined with usual care compared to conventional treatment combined with usual care (RR = 1.63, 95%CI: [0.74, 3.58], *p* = 0.23, I^2^ = not applicable) (Figure 9). Additionally, when conducting subgroup analysis by patient age, there were statistically significant differences in the cure rate of AA among different age groups (Table 2).

##### 3.4.2.3 PAC-QOL

**FIGURE 10:**
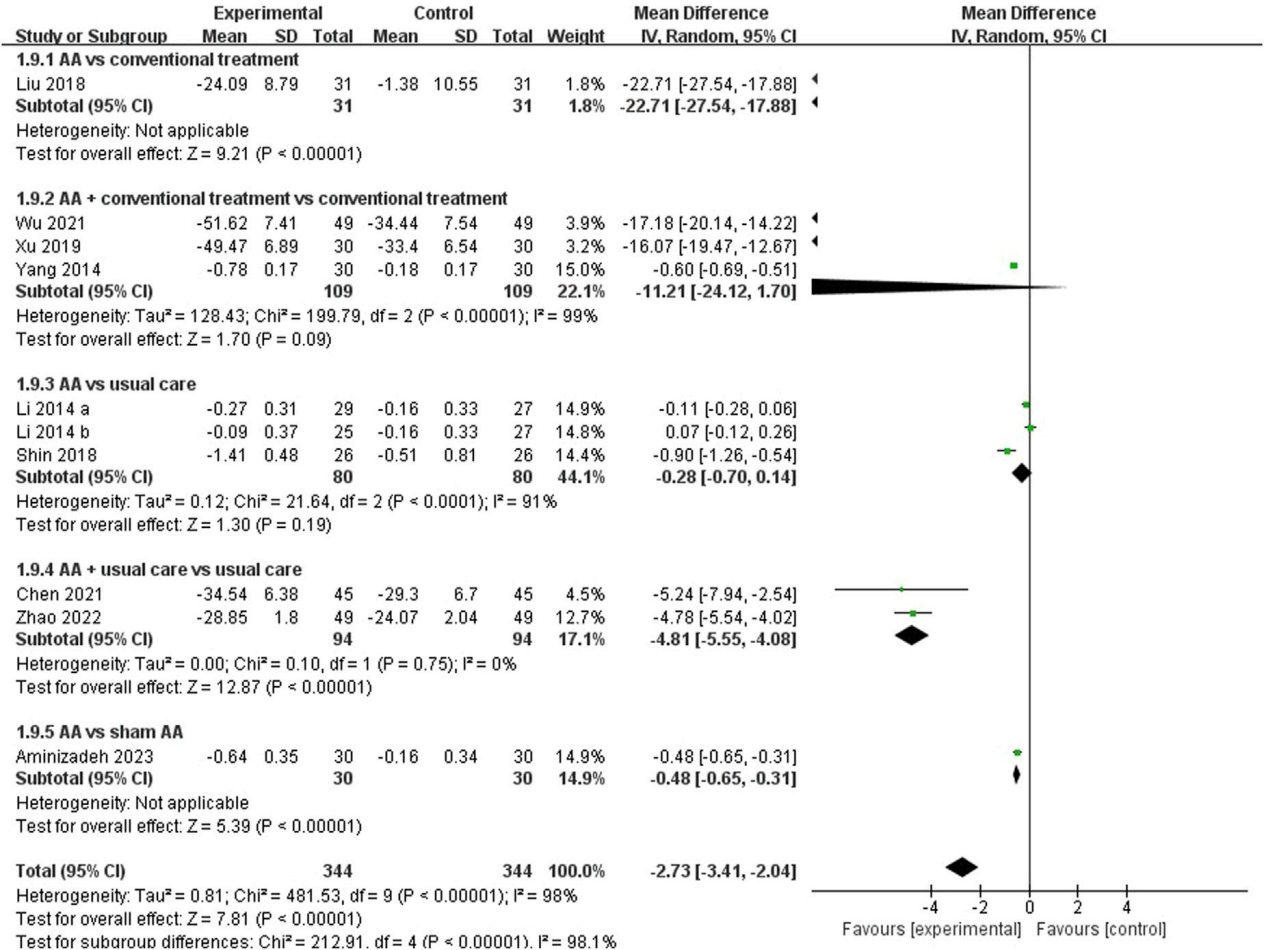
Meta-analysis and forest plot for PAC-QOL.

Nine studies (Li et al., 2014; Yang et al., 2014; Liu, 2018; Shin and Park, 2018; Xu, 2019; Chen et al., 2021; Wu et al., 2021; Zhao and Wang, 2022; Aminizadeh et al., 2023) reported the PAC-QOL. The results meta-analysis showed a greater improvement in the experimental group compared to the control group (MD = −2.73, 95% CI: [-3.41, −2.04], *p* < 0.00001, I^2^ = 98%). A subgroup analysis was performed according to different comparators, the results showed there was no significant improvement in PAC-QOL between AA combined with conventional treatment and conventional treatment (MD = −11.21, 95% CI: [-24.12,1.70], *p* = 0.09, I^2^ = 99%). Given the significant heterogeneity, a sensitivity analysis was employed. When removing the article by Yang et al. (Yang et al., 2014) the heterogeneity decreased from 99% to 0%. Additionally, there was no significant improvement between AA and usual care groups (MD = −0.28, 95% CI: [-0.70, 0.14], *p* = 0.19, I^2^ = 91%). However, AA was superior to conventional treatment (*p* < 0.00001) or sham AA (*p* < 0.00001) in terms of PAC-QOL, furthermore, the decrease in PAC-QOL score was more significant in the AA combined with usual care group (MD = −4.81, 95%CI: [-5.55, −4.08], *p* < 0.00001, I^2^ = 0%) than in the usual care group (Figure 10). Additionally, when performing subgroup analysis based on patient age, except for the elderly group aged 60 and above, AA showed statistical significance in improving PAC-QOL for constipated patients (Table 2).

##### 3.4.2.4 Adverse events

**FIGURE 11:**
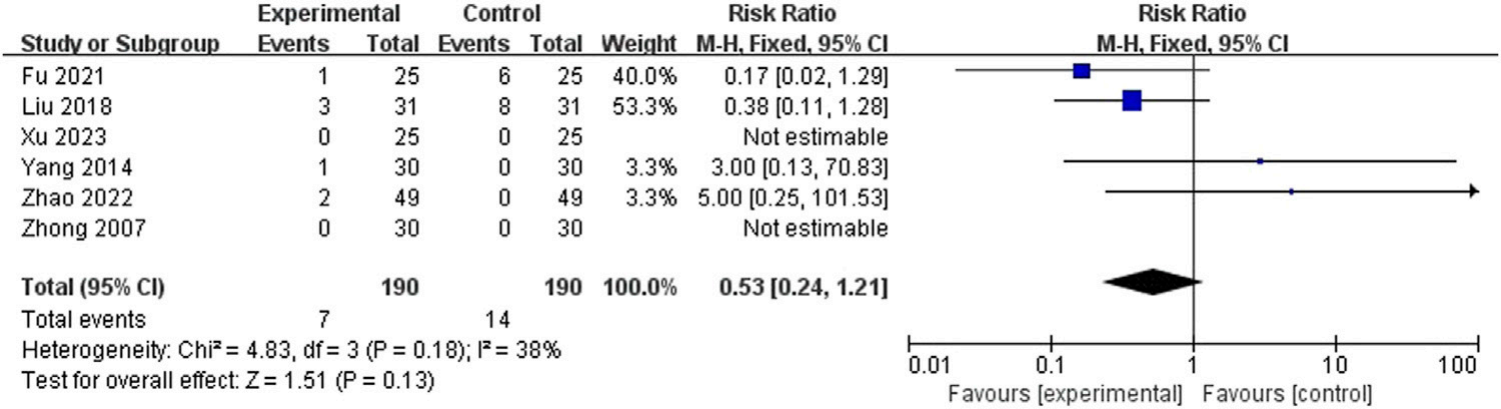
Meta-analysis and forest plot for adverse events.

A total of seven articles (Zhong, 2007; Yang et al., 2014; Liu, 2018; Fu et al., 2021; Zhao and Wang, 2022; Aminizadeh et al., 2023; Xu, 2023) mentioned adverse events, of these, one study (Aminizadeh et al., 2023) could not be included for meta-analysis due to poor description. The results of meta-analysis showed AA alone or in combination with other treatments was equally safe compared to other treatments (RR = 0.53, 95% CI: [0.24, 1.21], *p* = 0.13, I^2^ = 38%) (Figure 11). The adverse reactions were mostly minor, such as skin lesions or itching at the site of pressure, which typically resolved within a few days.

### 3.5 Quality of evidence

Table 3 presents the quality evaluation of each outcome of AA on constipation. The GRADE system was used to assess the evidence quality based on different intervention measures. The results revealed one outcome with moderate-quality evidence, nine with low-quality evidence, seven with very low-quality evidence, and no evidence with high quality. Most studies had limitations in their experimental design, such as inadequate description of random sequence generation, allocation concealment, and blinding. Consequently, they were downgraded. Additionally, the presence of publication bias in most studies contributed to their downgrading.

**TABLE 3:** Quality of evidence included RCTs by GRADE.

Outcomes	Number	T/C	End of treatment				
			F/R	MD/RR and 95%CI	Heterogeneity	*p*-value	GREDA
PAC-SYM							
AA vs. sham AA	2	58/59	R	MD = -3.04, 95% CI: [-0.49, −0.19]	I^2^ = 0%	*p* < 0.0001	Low
AA vs. usual care	2	54/54	R	MD = -0.04, 95%CI: [-0.240.15]	I^2^ = 38%	*p* = 0.68	Low
CSBMs	2	69/69	F	MD = 1.22, 95% CI: [0.68, 1.77]	I^2^ = 0%	*p* < 0.0001	Low
BSF	4	123/124	R	MD = 0.72, 95% CI: [0.15, 1.28]	I^2^ = 82%	*p* = 0.01	Very low
CAS	2	52/53	R	MD = -3.28, 95%CI: [-5.95, −0.60]	I^2^ = 80%	*p* = 0.02	Very low
Responder rate							
AA vs. conventional treatment	7	254/253	R	RR = 1.24, 95%CI: [1.11,1.39]	I^2^ = 39	*p* = 0.0002	Low
AA plus conventional treatment vs. conventional treatment	6	219/219	R	RR = 1.23, 95%CI: [1.04, 1.46]	I^2^ = 74%	*p* = 0.02	Very low
AA vs. usual care	3	117/115	R	RR = 1.28, 95%CI: [0.99, 1.66]	I^2^ = 78%	*p* = 0.06	Very low
AA+ usual care vs. usual care	8	294/294	R	RR = 1.34, 95%CI: [1.05, 1.70]	I^2^ = 92%	*p* = 0.02	Very low
Cure rate							
AA vs. conventional treatment	6	211/210	F	RR = 2.68, 95% CI [1.68, 4.28]	I^2^ = 0%	*p* < 0.0001	Moderate
AA+ usual care vs. usual care	7	245/245	F	RR = 1.79, 95% CI [1.34, 2.38]	I^2^ = 0%	*p* < 0.0001	Low
AA vs. usual care	2	77/75	F	RR = 1.84, 95% CI [1.30, 2.62]	I^2^ = 0%	*p* = 0.0007	Low
AA plus conventional treatment vs. conventional treatment	6	219/219	F	RR = 1.59, 95% CI [1.21, 2.09]	I^2^ = 0%	*p* = 0.0008	Low
PAC-QOL							
AA+ usual care vs. usual care	2	94/94	R	MD = −4.81, 95%CI: [-5.55, −4.08]	I^2^ = 0%	*p* < 0.00001	Low
AA vs. usual care	3	80/80	R	MD = −0.28, 95% CI: [-0.70, 0.14]	I^2^ = 91%	*p* = 0.19	Very low
AA plus conventional treatment vs. conventional treatment	3	109/109	R	MD = −11.21, 95% CI: [-24.12, 1.70]	I^2^ = 99%	*p* = 0.09	Very low
Adverse events	6	190/190	F	RR = 0.53, 95% CI: [0.24, 1.21]	I^2^ = 38%	*p* = 0.13	Low

MD, weighted mean difference; RR, risk ratio; CI, confidence interval; PAC-SYM, Patient Assessment of Constipation-Symptom; CSBMs, Complete spontaneous bowel movements; BSF, bristol stool form; CAS, constipation assessment scale; PAC-QOL, Patient Assessment of Constipation-Quality of Life questionnaires; AA, auricular acupressure; P, *p*-value represents clinical significance; T, test group; C, control group; F, fixed effects model; R, random effects model; GRADE, grades of recommendation, Assessment, Development, and Evaluation; Very low, very low quality; Low, low quality; Moderate, Moderate quality.

### 3.6 Reporting bias

**FIGURE 12:**
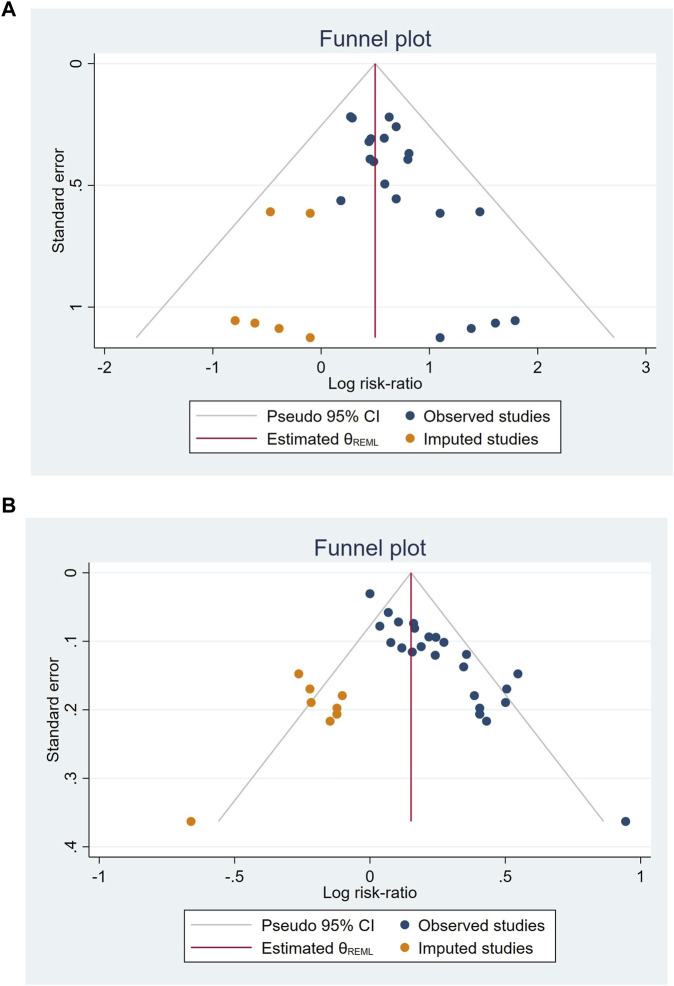
Funnel plots of included studies on responder rate **(A)**, cure rate **(B)**.

**FIGURE 13:**
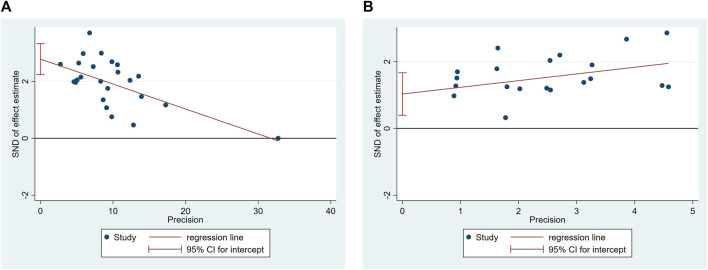
Egger’s test of included studies on responder rate **(A)**, cure rate **(B)**.

Due to the limited number of studies reporting outcome results, funnel plots and Egger’s tests were conducted only for response rate and cure rate. The funnel plot of included studies on responder rate displays an asymmetrical distribution, with four studies lying outside the pseudo 95% CI, indicating significant publication bias (Figure 12A). The funnel plot of included studies on cure rate indicates a roughly symmetrical distribution, suggesting a certain degree of publication bias and the presence of a small sample size effect (Figure 12B). Egger’s tests revealed significance levels of *p* < 0.05 for both response rate (*p* = 0.00) and cure rate (*p* = 0.003) (Figure 13).

## 4 Discussion

Constipation, a prevalent gastrointestinal condition, is characterized by persistent difficulties in bowel movements, incomplete defecation, and decreased frequency of bowel movements. This condition significantly impacts patients’ daily lives and can impair social functioning, leading to work or education limitations in a considerable proportion of individuals (Rao et al., 2007). Initial management strategies for constipation in both children and adults typically involve non-pharmacological approaches, as they have demonstrated beneficial effects in many cases (Daniali et al., 2020; Vriesman et al., 2020). AA, a non-invasive and non-pharmacological treatment modality, has gained widespread utilization for managing various chronic conditions, including constipation. Several studies have reported positive outcomes of AA in alleviating constipation symptoms (Li et al., 2014; Shin and Park, 2018; Jung and Chang, 2020; Aminizadeh et al., 2023). However, the safety and efficacy of AA for constipation in adults remain uncertain.

As is widely known, SRs and meta-analyses generate the highest level of clinical evidence (Izzo et al., 2016). Therefore, we conducted this study to assess the efficacy and safety of AA for constipation in adults. Overall, our study revealed statistically significant differences between the AA and control groups in terms of CSBMs, BSF, CAS, PAC-QOL, responder rate and cure rate, whereas it showed no statistical difference in PAC-SYM. However, when comparing AA with sham AA, AA could significantly reduce the PAC-SYM score (MD = -3.04, 95% CI: [-0.49, −0.19], *p* < 0.0001). These findings suggest that AA has the potential to alleviate constipation symptoms, increase the frequency of spontaneous bowel movements, improve stool consistency, and enhance overall quality of life. These results are consistent with previous studies examining the effects of AA on constipation in leukemia patients undergoing chemotherapy (Chen et al., 2018a; Jing et al., 2018a). Adverse events reported in seven studies were primarily mild, such as itching at the application site, with no serious adverse reactions documented.

It is worth noting that various outcome measures have been employed to assess the efficacy of constipation treatments. Some measures focus on single-item scores related to constipation (e.g., CSBMs), while others provide a comprehensive evaluation of different aspects of constipation (e.g., CAS). However, certain measures may not precisely reflect specific treatment efficacy, such as response rate or cure rate. To ensure the selected measures adequately capture the effectiveness of constipation treatments, future studies are recommended to include CSBMs per week as one of the outcome measures. This measure provides a straightforward and intuitive reflection of treatment effectiveness and has been widely adopted in recent high-quality clinical studies (Camilleri et al., 2022; Rao et al., 2023; Wang et al., 2023). In our study, AA was found to increase the number of complete spontaneous bowel movements [MD = 1.22, 95% CI (0.68, 1.77), *p* < 0.0001], with low heterogeneity (I^2^ = 0%).

AA, derived from traditional Chinese Medicine (acupuncture), focuses on manipulating specific acupoints on the ear to achieve therapeutic effects. This technique is based on the concept of Qi, the vital energy flow within the body. Through applying pressure to these acupoints, practitioners aim to regulate Qi and restore balance (Yang et al., 2013). During auricular acupressure, individuals may experience sensations such as soreness, tingling, or numbness, indicating the activation of acupoints and the desired therapeutic response. Among the included studies, only one study (Li et al., 2014) did not require pressure application to achieve the intended stimulation of Qi. In most other studies, participants were instructed to apply pressure 3–5 times daily. This practice is widely accepted in China (Bao et al., 2017). Furthermore, based on the included research findings, AA involves applying pressure to the acupoints on one side at a time and alternating between both sides. However, there is considerable variation in treatment duration among the studies, ranging from 1 week to 8 weeks. Further research is needed to evaluate the optimal duration and effectiveness of AA. The selection of ear acupoints primarily follows the TCM *Zang-fu* theory (Liu et al., 2021). The most frequently used acupoints include the Large Intestine and Rectum points to increase intestinal peristalsis, the Spleen point to strengthen transportation and transformation functions, and the San Jiao point to regulate the flow of qi and fluid in the body. When these points are used together, they are believed to promote smooth qi flow in the Large Intestine, ensuring normal transmission function.

However, it is important to acknowledge the limitations of this study. The variations in selected acupoints, comorbid conditions, treatment courses, and number of compressions may introduce significant clinical heterogeneity. Additionally, the restriction to English and Chinese clinical trials may introduce publication bias, as only four out of the 35 included studies were published in English. Most studies did not provide specific details regarding randomization, allocation concealment, and blinding methods, and some failed to comprehensively report trial details according to standard reporting guidelines such as CONSORT. As a result, the quality of evidence for the majority of outcomes was deemed low or very low.

## 5 Conclusion

Our findings suggest that AA may be a safe and effective treatment for constipation in adults to improving symptoms of constipation and increasing the frequency of spontaneous bowel movements. Nevertheless, it is important to note that the current evidence quality is considered insufficient, and further validation is required through a greater number of high-quality RCTs.

The author(s) declare that no financial support was received for the research, authorship, and/or publication of this article.

## Data Availability

The original contributions presented in the study are included in the article/Supplementary Material, further inquiries can be directed to the corresponding author.
