# Supplementary material for: Auricular acupressure for constipation in adults: a systematic review and meta-analysis
Source: Front Physiol. 2023 Oct 16;14:1257660. doi: 10.3389/fphys.2023.1257660 (PMC10613998; doi:10.3389/fphys.2023.1257660)
Supplement: Supplementary file 1 [file Table2.DOCX]

Supplementary Material

Article Title

First Author: Ze-fei Jiang

*** Correspondence:** Hong Zhang : [hzhang0123@foxmail.com](mailto:hzhang0123@foxmail.com)

# Supplementary Data

# Pubmed:

(((constipation[MeSH Terms]) OR ((((constipation[Title/Abstract]) OR (astriction[Title/Abstract])) OR (dyschezia[Title/Abstract])) OR (obstipation[Title/Abstract]))) AND ((Acupressure[MeSH Terms]) OR ((((((auriculotherapy[Title/Abstract]) OR (auricular acupressure[Title/Abstract])) OR (Acupunctures, Ear[Title/Abstract])) OR (Ear Acupuncture[Title/Abstract])) OR (Auricular Acupuncture[Title/Abstract])) OR (Acupuncture, Auricular[Title/Abstract])))) AND ((((Randomized Controlled Trials as Topic[MeSH Terms]) OR (random allocation[MeSH Terms])) OR ((((randomized[Title/Abstract]) OR (randomly[Title/Abstract])) OR (RCT[Title/Abstract])) OR (trial[Title/Abstract]))) OR ((Randomized Controlled Trial[Publication Type]) OR (clinical trial[Publication Type])))

Results:26

**Web of Science:**

(Topic =Constipation OR functional constipation OR abstriction OR dyschezia OR obstipation) AND (Topic =Randomized Controlled Trials as Topic OR random allocation OR randomized OR randomly OR RCT OR trial OR Randomized Controlled Trial OR clinical trial) AND (Topic =Acupressure OR auriculotherapy OR auricular acupressure OR acupunctured, Ear OR Ear Acupuncture OR Auricular Acupuncture OR Acupuncture, Auricular)

Results:34

**Embase:**

(constipation:ab,ti OR 'functional constipation':ab,ti OR abstriction:ab,ti OR dyschezia:ab,ti OR obstipation:ab,ti) AND ('randomized controlled trials as topic':ab,ti OR 'random allocation':ab,ti OR randomized:ab,ti OR randomly:ab,ti OR rct:ab,ti OR trial:ab,ti OR 'randomized controlled trial':ab,ti OR 'clinical trial':ab,ti) AND (acupressure:ab,ti OR auriculotherapy:ab,ti OR 'auricular acupressure':ab,ti OR 'acupunctured, ear':ab,ti OR 'ear acupuncture':ab,ti OR 'auricular acupuncture':ab,ti OR 'acupuncture, auricular':ab,ti)

Results:37

**CENTRAL:**

(Title Abstract Keyword =Constipation OR functional constipation OR abstriction OR dyschezia OR obstipation) AND (Title Abstract Keyword =Randomized Controlled Trials as Title Abstract Keyword OR random allocation OR randomized OR randomly OR RCT OR trial OR Randomized Controlled Trial OR clinical trial) AND (Title Abstract Keyword =Acupressure OR auriculotherapy OR auricular acupressure OR acupunctured, Ear OR Ear Acupuncture OR Auricular Acupuncture OR Acupuncture, Auricular)

Results:60

**CNKI:**

主题=（耳穴按压 OR 耳穴贴压 OR 耳穴点按 OR 耳穴压丸 OR 耳穴压豆 OR 耳穴埋豆 OR 耳穴埋籽）AND 主题=（便秘 OR 排便困难 OR 大便困难）

Results:610

**WanFang:**

主题=（耳穴按压 OR 耳穴贴压 OR 耳穴点按 OR 耳穴压丸 OR 耳穴压豆 OR 耳穴埋豆 OR 耳穴埋籽）AND 主题=（便秘 OR 排便困难 OR 大便困难）

Results:911

**VIP:**

篇名或关键词=（耳穴按压 OR 耳穴贴压 OR 耳穴点按 OR 耳穴压丸 OR 耳穴压豆 OR 耳穴埋豆 OR 耳穴埋籽）AND 篇名或关键词=便秘

Results:534

**CBM:**

( "耳穴按压"[标题] OR "耳穴贴压"[标题] OR "耳穴点按"[标题] OR "耳穴压丸"[标题] OR "耳穴压豆"[标题] OR "耳穴埋豆"[标题] OR "耳穴埋籽"[标题]) AND "便秘"[标题]

Results:417
